# Identifying clinical and psychological characteristics of cardiac surgery patients

**DOI:** 10.1192/j.eurpsy.2022.1165

**Published:** 2022-09-01

**Authors:** O. Nikolaeva, T. Karavaeva, E. Nikolaev, S. Petunova, N. Grigorieva, E. Lazareva

**Affiliations:** 1 Ulianov Chuvash State University, Department Of Faculty And Hospital Therapy, Cheboksary, Russian Federation; 2 Republican Cardiology Clinic, Cardiosurgery Unit, Cheboksary, Russian Federation; 3 St. Petersburg State University, Department Of Medical Psychology And Psychophysiology, St. Petersburg, Russian Federation; 4 V. M. Bekhterev National Medical Research Center for Psychiatry and Neurology, Department For Treatment Of Borderline Mental Disorders And Psychotherapy, St. Petersburg, Russian Federation; 5 St. Petersburg State Pediatric Medical University, Department Of General And Applied Psychology With A Course In Biomedical Disciplines, St. Petersburg, Russian Federation; 6 N.N. Petrov National Medical Research Center of Oncology, Scientific Department Of Innovative Methods Of Therapeutic Oncology And Rehabilitation, St. Petersburg, Russian Federation; 7 Ulianov Chuvash State University, Social And Clinical Psychology Department, Cheboksary, Russian Federation

**Keywords:** hostility, depressive disorders, cardiac surgery patients, time perspective

## Abstract

**Introduction:**

Cardiac surgery patients (CSP) are cardiovascular patients who undergo surgery to treat their disease. Are their psychological characteristics different from those of other cardiac patients?

**Objectives:**

The goal is to establish peculiarities of the clinical-and-psychological status of CSPs in different clinical groups.

**Methods:**

According to clinical parameters, 152 CSPs were divided into three groups. The first group comprised patients with CHD indicated to an open-heart coronary artery bypass grafting, the second one included patients with heart failure who were to undergo aortic valve surgery, and the third group included CHD patients and those with heart rhythm abnormalities indicated to minimally invasive surgery.

**Results:**

CSPs had a number of cardiologic complaints, mental disturbance manifestations and concomitant somatic diseases. They showed difference in the duration of the disease, previous occurrence of heart surgery or myocardial infarction, and in the degree of heart failure manifestations. Self-assessment of pre-surgery CSPs corresponded to the severity of their clinical condition, while indications of hope for recovery were at the maximum level. The second group showed a moderate level of depression, while the third one – slight depression. All the groups revealed a disharmonic profile of time perspective. Group 1 CSPs showed some manifestations of hostility. We saw different manifestations of CSPs’ personal adaptation resources. While hardiness had insufficient showings at the level of most components, social support was excessive in all groups.

**Conclusions:**

CSPs as other cardiac patients revealed depressive disorders and hostility. At the same time, they have more social support, which testifies availability of good interpersonal resources.

**Disclosure:**

No significant relationships.

